# Using genomic information for management planning of an endangered perennial, *Viola uliginosa*

**DOI:** 10.1002/ece3.6093

**Published:** 2020-02-17

**Authors:** Kyung Min Lee, Pertti Ranta, Jarmo Saarikivi, Lado Kutnar, Branko Vreš, Maxim Dzhus, Marko Mutanen, Laura Kvist

**Affiliations:** ^1^ Ecology and Genetics Research Unit University of Oulu Oulu Finland; ^2^ Faculty of Biological and Environmental Sciences University of Helsinki Helsinki Finland; ^3^ Department of Forest Ecology Slovenian Forestry Institute Ljubljana Slovenia; ^4^ Jovan Hadži Institute of Biology ZRC SAZU Ljubljana Slovenia; ^5^ Department of Botany Belarusian State University Minsk Belarus

**Keywords:** conservation, demography, genomic diversity, population genomics, RAD sequencing, *Viola*

## Abstract

Species occupying habitats subjected to frequent natural and/or anthropogenic changes are a challenge for conservation management. We studied one such species, *Viola uliginosa*, an endangered perennial wetland species typically inhabiting sporadically flooded meadows alongside rivers/lakes. In order to estimate genomic diversity, population structure, and history, we sampled five sites in Finland, three in Estonia, and one each in Slovenia, Belarus, and Poland using genomic SNP data with double‐digest restriction site‐associated DNA sequencing (ddRAD‐seq). We found monophyletic populations, high levels of inbreeding (mean population *F*_SNP_ = 0.407–0.945), low effective population sizes (*N*_e_ = 0.8–50.9), indications of past demographic expansion, and rare long‐distance dispersal. Our results are important in implementing conservation strategies for *V. uliginosa*, which should include founding of seed banks, ex situ cultivations, and reintroductions with individuals of proper origin, combined with continuous population monitoring and habitat management.

## Graphical abstract

We investigated endangered and nonendangered populations of a violet, *Viola uliginosa*, to estimate genomic diversity, population structure, and history from genomic data. We found monophyletic populations, high levels of inbreeding, and low effective population sizes and indications of past demographic expansion and rare long‐distance dispersal. These are proposed to include the founding of seed banks, ex situ cultivations, and reintroductions with individuals of proper origin, combined with continuous population monitoring and habitat management.

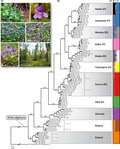

## 1 INTRODUCTION

Endangered species inhabiting patchy, periodically changing habitats are especially challenging for conservation efforts. Populations of such species have a high risk of extinction simply due to environmental stochasticity, and if populations are sparse, as is usually the case with endangered species, recolonizations of locally extinct populations will be unlikely. The temporal extinction–recolonization dynamics, that is, metapopulation dynamics, has been shown to depend on the number of available habitats, extinction rate, and colonization rate. However, the classic metapopulation model (e.g., Hanski, 1998; Levins, 1969) assumes that the dynamics of a species is greater than stability of the habitat patches. Thus, species occupying patchy and rapidly changing habitats must use habitat tracking for successful colonization (Thomas, 1994).

Colonization of a new habitat exposes the population to a founder effect, or a population bottleneck due to a small number of colonizing and often related individuals. As the founding population represents only a part of the source population, it most likely will be genetically differentiated from the source at emergence. For example, riparian populations of oregano (*Origanum vulgare*; Van Looy, Jacquemyn, Breyne, & Honnay, 2009) and roadside populations of white campion (*Silene latifolia*; Fields & Taylor, 2014) display strong genetic differentiation between populations due to founder effects. A small founding population also has a small effective population size, which combined with genetic drift can lead to further genetic differentiation between newly founded populations and their source population. This was illustrated in the annual jewelweed (*Impatiens capensis*), which showed strong genetic structuring along bodies of water, due mainly to genetic drift (Toczydlowski & Waller, 2019). Additionally, differential selection pressures can increase divergence between the populations at the newly occupied and the source sites, if the newly occupied site stays occupied long enough for natural selection to operate. For instance, the endangered Spanish catchfly (*Silene otites*), in situ, and their respective ex situ populations became highly genetically differentiated within a few decades, partly attributed not only to the effect of genetic drift, but also to unintended selection during cultivation (Lauterbach, Burkart, & Gemeinholzer, 2012).

Genetic diversity, therefore, tends to be lost during founding of new populations. Such cases have been reported in an isolated population of alder buckthorn (*Frangula alnus*) in Ireland (Finlay, Bradley, Preston, & Provan, 2017) and in invasive populations of Carolina geranium (*Geranium carolinianum*) in China (Shirk, Hamrick, Zhang, & Qiang, 2014). Moreover, small populations commonly have lower genetic diversity than large populations, clearly demonstrated in a survey of 247 plant species, where rare and common species were compared at a generic level using allozymes and the rare species had significantly lower genetic diversity than their common counterparts (Cole, 2003). Further, small populations are prone to increased homozygosity, inbreeding, and inbreeding depression, leading to reduced fitness of highly related individuals (Charlesworth & Willis, 2009), which can threaten their viability (Frankham, 2005).

The above features pose challenges for conservation management. If a habitat for a population is lost for a period of time, a population could be reintroduced to the site provided that the habitat once again becomes favorable and a proper source population exists. When an imminent threat to an occupied habitat exists, the population could be relocated to another suitable site. Identification of proper source populations or introduction sites requires understanding of genetic structure and diversity of the species at the range in question, in addition to understanding the evolutionary processes underlying the observed genetic structure and diversity.

Here, we studied endangered populations of *Viola uliginosa*, which are at the northern edge of the species’ range in Finland, to clarify the amount of genetic diversity, inbreeding, and population structure. Our goal was to define whether and which populations could be used as source populations for reintroductions to sites from which the species became locally extinct, but are still suitable for *V. uliginosa*. In addition, we sampled nearby populations from Estonia and distant populations at the center and western edge of the distribution range to infer the origin and phylogenetic position of the Finnish populations.

## 2 MATERIALS AND METHODS

### 2.1 Study species and sampling

**Figure 1:**
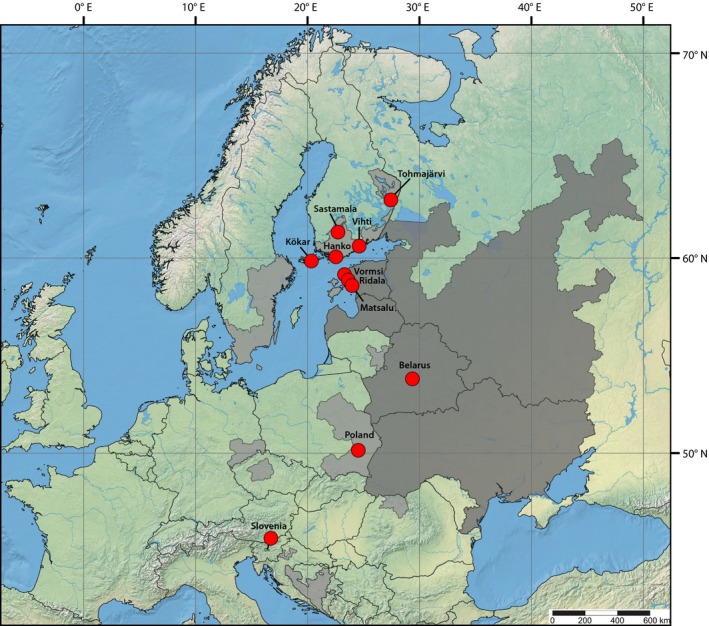
The updated distribution map of *Viola uliginosa* redrawn from Ranta et al. (2016) with slight modifications according to Matulevičiūté (2015) and the Swedish Species Information Centre (2019). The core areas are colored dark gray, and the peripheral areas, light gray. Red circles indicate sampling sites of this study

*Viola uliginosa* Besser is a perennial wetland species that inhabits typically rich, flooded meadows along rivers/lakes. The stronghold for the species distribution is in Eastern Europe, with sporadic sites at the western and northern edges of the main distribution (Figure 1). *Viola uliginosa* is listed as endangered in Finland (Ryttäri et al., 2019), existing at present in only six sites. It has been monitored for 30 years in an attempt to reduce further loss (Siitonen, 1990). As it occupies rare eutrophic swamp forests and flooded meadows, it can be considered as an indicator species for these habitats (Kuris & Ruskule, 2006). The species also has been classified as near‐threatened, vulnerable, endangered, or critically endangered in many other parts of its range, primarily because of the habitat type (e.g., temporal flooding mostly at springtime), habitat loss, and human disturbance (Ranta, Jokinen, & Laaka‐Lindberg, 2016).

*Viola uliginosa* reproduces primarily by clonal propagation using subterranean stems (Cieślak, Paul, & Ronikier, 2006; Paul et al., 2016), but it also forms seeds that are able to exist in a seed bank and can be viable for years (Ranta & Siitonen, 2011). Individual plants can develop both chasmogamous (CH, open flowers, enabling cross‐pollination) and cleistogamous (CL, closed flowers, resulting in obligatory self‐pollination) flowers, as well as intermediate forms (semi‐CL) (Małobęcki et al., 2016). Pollination in this species has not been studied in detail, but cross‐pollination occurs by several species of bees, hoverflies, and flies, as in other *Viola* species, in addition to self‐pollination (Beattie, 1971). Even though reproduction is thought to be mostly clonal, seeds have a high germination capacity, putatively allowing rapid adaptation to changes in the habitat (Ranta et al., 2016). Local‐level seed dispersal by snails and ants has been observed to occur, and long‐distance dispersal can occur by floating in water currents (P. Ranta, personal communication).

We focused our sampling on extant Finnish populations of *V. uliginosa*, which constitute the northern edge of its global distribution. For inferring the history of the Finnish populations, we also collected samples from Estonia (isolated by the Gulf of Finland) and Belarus, both of which form the species’ center of distribution and from Poland and Slovenia, which form the southern and western edge populations, respectively. Thus, our sampling provides a latitudinal perspective. We collected one or two leaves per individual from each sampling site during summer 2016 and 2017 (with all relevant permits), with a special effort to sample individuals as distantly from each other as possible from each site. We sampled five sites in Finland (Kökar, Åland *N* = 30, Hanko *N* = 30, Vihti *N* = 30, Sastamala *N* = 20, Tohmajärvi *N* = 30), three in Estonia (Maatsalu *N* = 21, Vormsi, two populations *N* = 10 + 20, Ridala *N* = 21), one in Slovenia (Ljubljansko Barje *N* = 30), one in Belarus (Iljinka, consisting of two adjacent sites, *N* = 10 + 30), and one in Poland (Lipa, consisting of three adjacent sites, *N* = 10 in all) (Figure 1). The sixth known Finnish site in Mäntsälä was rapidly declining at the time of sampling, thus was not sampled (see Table S1 for more detailed sampling information). Leaf samples were stored dry in plastic bags containing silica gel. DNA was extracted with DNeasy Plant Kit (Qiagen) or PowerPlant DNA Isolation Kit (Mobio) according to manufacturer's protocols. Based on the quality and quantity of obtained DNA, we chose 96 individuals (eight individuals per population except 16 for Vormsi) including one outgroup species (*V. mirabilis*) for further analyses (see Table S1).

### 2.2 ddRAD library preparation and sequencing

The quantity of genomic DNA (gDNA) was determined with the PicoGreen Kit (Molecular Probes, Eugene, OR, USA). The ddRAD‐seq library was implemented following protocols described in Peterson, Weber, Kay, Fisher, and Hoekstra (2012) and Lee et al. (2018) with the following modifications. Briefly, gDNA was digested at 37°C for 3 hr using the restriction enzymes *Pst*I and *Mse*I (NEB) followed by a ligation step, whereby each sample was assigned to one of 48 adaptors. Pools of 48 individuals were combined and run on 1.5% agarose cartridge in the automated size‐selection technology, BluePippin (Sage Science), where 300‐bp fragments were excised. Each pool was amplified using 16 PCR cycles in 50 µl reactions containing 30 µl Phusion High‐Fidelity PCR Master Mix (Thermo Fisher Scientific), 15 µl of library DNA, and a unique indexing primer for each pool that corresponds to the standard Illumina multiplexed sequencing protocol. PCRs were performed in a Veriti 96‐Well Thermal Cycler (Life Technologies) using the following protocol: initial step heating at 98°C for 30 s, 16 cycles (98°C for 10 s, 60°C for 30 s, and 72°C for 40 s), followed by a final step at 72°C for 5 min. DNA libraries were quantified using the High‐Sensitivity DNA Analysis Kit in a 2100 Bioanalyzer (Agilent Technologies). Pools were combined in equimolar concentration to form a single genomic library and sequenced in one lane of a HiSeq 2500 Illumina sequencer (paired‐end, v4 reagents). The demultiplexed *V. uliginosa* fastq data are archived in the NCBI SRA: PRJNA540749.

### 2.3 Bioinformatics

Raw paired‐end reads were demultiplexed with no mismatches tolerated using their unique barcode and adapter sequences with *ipyrad* v.0.7.23 (Eaton & Overcast, 2016). The quality of raw demultiplexed reads was checked with FastQC software (available at http://www.bionformatics.babraham.ac.uk/projects/fastq/). The demultiplexed paired‐end reads were run through PEAR (Zhang, Kobert, Flouri, & Stamatakis, 2014) using default setting to merge overlapping reads, and input into the *ipyrad* pipeline. All *ipyrad* defaults were used, with the following exceptions: The minimum depth at which majority rule base calls are made was set to 3, the cluster threshold was set to 0.90, the minimum number of samples that must have data at a given locus for it to be retained was set to 48, 70, and 95, and the assembly method was set to “denovo” and “reference” for independent testing. Consensus sequences that had low coverage (<6 reads), excessively undetermined, or characterized with many heterozygous sites (>8), potentially resulting from paralogs or highly repetitive genomic regions, were discarded. Additionally, we excluded all loci with excessive (>50% of samples) shared polymorphic sites as they likely represented clustering of paralogs. Up to four shared polymorphic sites per called locus were allowed to accommodate polyploid genomes. This was done, because tetraploid marker data can be indistinguishable from diploid data (Gompert & Mock, 2017) and the species has been reported to produce polyploid individuals in a micropropagation experiment (Slazak et al., 2015); thus to our knowledge, polyploidy has not been observed in the wild. As there was no evidence of polyploidy, all samples were treated as diploids in the following analyses, thus allowing two haplotypes per polymorphic site. In the “denovo” assembly, sequences were assembled without any reference genome with homology inferred during alignment clustering by sequence similarity using the program *vsearch* (http://github.com/torognes/vsearch). In the “reference” assembly, the sequences were mapped to the whole genome of *Viola pubescens* (GenBank, GCA_002752925) using *BWA* with default *bwa‐mem* setting (Li, 2013) based on 90% of sequence similarity.

Phylogenetic trees were generated for “ddrad_m48” data matrices (Table 1) using a maximum‐likelihood method in RAxML v.8.2.0 (Stamatakis, 2014) with node supports estimated by a 1,000 rapid bootstrap replicates based on aligned concatenated sequences (with the following commands: ‐f v ‐m GTRCAT). One individual sampled from Tohmajärvi, identified as *V. mirabilis* based on BLASTN results of the ddRAD sequences, was used as an outgroup. *Viola mirabilis* belongs to the same subgroup *Rostratae* than *V. uliginosa* and these two species have even been suggested as sister species, making it a good species to be used as an outgroup (van den Hof, Berg, & Gravendeel, 2008; Małobęcki et al., 2016). The resulting phylogeny was visualized using FigTree v.1.4.2 (Rambaut, 2015).

**Table 1:** Summary statistics of ddRAD datasets

Data matrix	ddrad_m48	ddrad_m95	ref_ddrad
Assembly method	De novo	De novo	Reference^a^
Loci	11,398	2,273	14,495
SNPs	31,724	4,143	51,435
Parsimony informative	8,770	1,713	21,405
Alignment length (bp)	2,040,210	432,991	2,512,418
Missing (%)	15.0	2.0	71.8

a *Viola pubescens* genome (GCA_002752925) was used as reference.

### 2.4 Genetic differences among populations

The ddrad_m95 dataset (Table 1) was used for population analysis. Patterns of genetic diversity among populations were examined using Arlequin v.3.5.1 (Excoffier & Lischer, 2010). The analyses were calculated on a per‐locus basis, using the consensus SNP set. To avoid possible biases due to low coverage in estimating nucleotide diversity, one haplotype for each individual was randomly sampled, and calculations were performed using this haploid subset. Pairwise *F*_ST_ values and nucleotide diversity (*π*) for all populations were calculated. AMOVA (analysis of molecular variance) quantified the proportion of variation at each organizational level based on unlinked SNP data with 1,000 permutations. All five Finnish populations were grouped into one group, the three Estonian populations into another group, and Belarusian, Polish, and Slovenian populations were retained as separate groups. Reasoning for the grouping was that Finnish and Estonian populations are separated by the Gulf of Finland, likely a strong dispersal barrier, and the Central European populations locate far apart. The grouping also reflects latitudinal locations of the study populations. Isolation by distance was tested using the Mantel test implemented in Arlequin, with Slatkin's linearized *F*_ST_ and natural logarithms of geographic distances.

SNP‐based inbreeding coefficients were calculated using vcftools (Danecek et al., 2011). Vcftools calculates the inbreeding coefficient *F*_SNP_ per individual using the equation *F*_SNP_ = (*O* – *E*) / (*N* – *E*), where *O* is the observed number of homozygotes, *E* is the expected number of homozygotes (given population allele frequency), and *N* is the total number of genotyped loci. Boxplots between populations and *F*_SNP_ values were executed with R v.3.5.2 (R Core Team, 2015) and graphically represented using the packages *corrplot* (Wei, 2013) and *ggplot2* (Wickham, 2009).

### 2.5 Clustering analysis

Population clustering with admixture from SNP frequency data was inferred to better visualize genomic variation between individuals with STRUCTURE v.2.3.1 (Pritchard, Stephens, & Donnelly, 2000). In this analysis, 1,958 putatively unlinked SNPs were identified by selecting a single SNP from each locus in the “ddrad_m95” data matrix (Table S1). Ten replicates were run with *K* = 1–11. Each run had a burn‐in of 50K generations followed by 500K generations of sampling. Replicates were permuted using CLUMPP (Jakobsson & Rosenberg, 2007) and the optimal *K* value was inferred using StructureHarvester (Earl & VonHoldt, 2012) according to the ad hoc Δ*K* statistics (Evanno, Regnaut, & Goudet, 2005), which is the second‐order rate of change in the likelihood function. Structure results were visualized using DISTRUCT (Rosenberg, 2004).

The package FineRADstructure was also used to investigate the genetic structure in *V. uliginosa* (Malinsky, Trucchi, Lawson, & Falush, 2018). The package includes RADpainter, a program designed to infer the co‐ancestry matrix and estimate the number of populations within the dataset. The input file used was an allele.loci matrix (“ddrad_m48” = 15% of missing data) generated with *ipyrad* program. The allele data were converted using a python script (available at http://github.com/edgardomortiz/fineRADstructure-tools; last accessed 21 June 2019). Samples were assigned to populations using 100,000 iterations as burn‐in prior to sampling with 100,000 iterations. The trees were constructed using 10,000 iterations and the output visualized using the fineradstructureplot.r and finestructurelibrary.r R scripts (http://cichlid.gurdon.cam.ac.uk/fineRADstructure.html).

To generate an unrooted genetic network, Neighbor‐Net analysis (Bryant & Moulton, 2003) was implemented in SplitsTree v.4.14.2 (Huson & Bryant, 2006) using uncorrected *p*‐distances with heterozygous ambiguities averaged and normalized with 1,000 bootstrap replicates (>75% shown). This method uses aspects of Neighbor‐Joining (Saitou & Nei, 1987) and SplitsTree to create a network that visualizes multiple hypotheses simultaneously.

### 2.6 Population history

Population demographic changes and deviation from neutrality were estimated based on mismatch distribution (Excoffier, 2004), the raggedness index (Harpending, 1994), and Tajima's D (Tajima, 1989) using Arlequin v.3.5.1 (Excoffier & Lischer, 2010). Mismatch distributions were constructed from the full dataset and based on combined Finnish and Estonian population to attain a sufficient sample size.

Effective population sizes (*N*_e_) for the contemporary and historical samples were calculated using the bias‐corrected measure of linkage disequilibrium (Waples & Do, 2010), as implemented in NeEstimator v.2.1 (Do et al., 2014) based on allele frequencies of all 4,143 loci. We estimated *N*_e_ for each population using minor allele frequency cutoff of 0.05.

**Figure 2:**
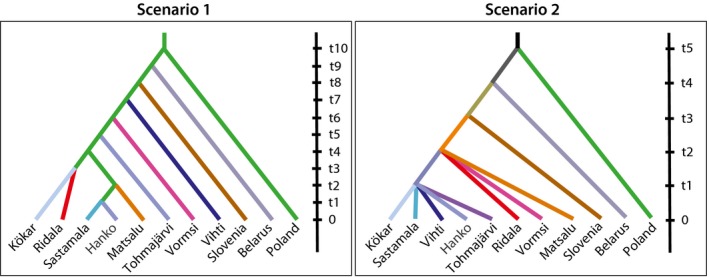
Scenarios used for DIYABC analysis

History was further evaluated using the program DIYABC v.2.0.3 (Cornuet et al., 2014), based on approximate Bayesian computation (ABC). Two different historical scenarios were compared, based on (a) branching order of the phylogenetic tree and (b) an otherwise similar branching order, but a simultaneous divergence of Estonian populations followed by a simultaneous divergence of the Finnish populations (Figure 2). After preliminary runs, the following priors were set: present effective population size (*N*_e_) for Poland, Slovenia, and Belarus of 10–1,000, and for other populations of 10–100, ancient population sizes of 10–10,000 for all and divergence times (*t*) of 10–1,000 for all. We ran 200,000 simulations with this scenario and used the 2,000 sets closest to the observed data for parameter estimation.

## 3 RESULTS

We obtained over 2,000,000‐bp sequence and 31,000 SNPs for the ddrad_m48 dataset and over 430,000‐bp sequence and over 4,100 SNPs for the ddrad_m95 dataset with only 15% and 2.0% of missing data, respectively. There were 8,770 parsimony‐informative sites in ddrad_m48 data and 1,713 in the ddrad_m95 data. The alignment with the reference sequence exceeded 2,500,000 bp and 51,400 SNPs (Table 1).

Nucleotide diversities ranged from 0.013 (Vihti) to 0.023 (Ridala). The three sites with lowest diversity values were all from Finland (Vihti, Kökar, and Hanko) (Table 2). Individual inbreeding coefficients were all positive and mostly very high. Out of the 39 Finnish individuals, 36 had inbreeding coefficients > 0.500, with 20 individuals above 0.900. One exceptionally low value of 0.084 was found for one individual from Tohmajärvi (range: 0.084–0.982). Of the 32 individuals from Estonia, 21 had values > 0.500, with two individuals having a value below 0.200 (range: 0.100–0.836). Inbreeding coefficients in Belarus were 0.386–0.741, in Poland 0.263–0.777, and in Slovenia 0.226–0.626. The average inbreeding coefficients of the studied populations were highest in the Finnish populations, with the most extreme value (0.945) obtained from the island population of Kökar (Figure S1 and Table 3). All populations had small effective population sizes based on NeEstimator, some even extremely small (Tohmajärvi 0.8, Poland 1.5). The largest effective population sizes were in two Estonian populations (Matsalu 50.9, Ridala 38.9) (Table 3).

**Table 2:** Diversity measures, Tajima's *D,* and mismatch distribution parameter estimates

Population	*n*	*S*	π	Tajima's *D*	H*Rag*	*τ*	*θ*_0_	*θ*_1_
Belarus	8	685	0.02042	−1.64723**	0.0714	76.000	9.429	1,000
Poland	8	430	0.01906	−1.44254*	0.0816	54.992	5.972	1,000
Slovenia	8	406	0.02113	−1.99967**	0.0587	50.410	8.268	1,000
Matsalu	8	695	0.01842	−2.15492**	0.0561	68.103	6.861	1,000
Ridala	8	756	0.02339	−1.13695	0.1327	86.079	5.635	1,000
Vormsi	16	840	0.01877	−2.13757**	0.0114	58.170	7.905	1,000
Hanko	8	624	0.01586	−1.21846	0.0740	66.192	4.558	1,000
Kökar	8	660	0.01494	−1.43031*	0.0485	79.612	2.996	1,000
Sastamala	8	779	0.02253	−1.67537**	0.0893	78.904	11.132	1,000
Tohmajärvi	7	659	0.02068	−1.78349**	0.0680	73.870	11.225	1,000
Vihti	8	589	0.01301	−1.64673**	0.0842	54.742	9.579	1,000
All	95	1,579	0.04395	−1.66120*	0.0004	58.951	10.677	1,000

Note

*n* = number of individuals, *S* = number of polymorphic sites, *π* = nucleotide diversity, H*Rag* = raggedness statistic *r*, *τ* = 2 *µt*, where *µ* is the mutation rate and *t* is time in generations, *θ*_0_ = theta before population size change, *θ*_1_ = theta after population size change.

Significant *p*‐values are indicated by *p < .05; **p < .02.

**Table 3:** Average linkage disequilibrium (*r* ^2^), effective population sizes (*N*_e_) with 95% confidence intervals, and average inbreeding coefficients (*F*_SNP_) with standard deviations in brackets of *Viola uliginosa* populations. Note that infinite estimates of the effective population size are likely due to a correction for the sample size that is larger than *r^2^* rather than a truly infinite population size (Macbeth, Broderick, Buckworth, & Ovenden, 2013)

Population	*r* ^2^	*N*_e_	95% confidence interval	*F*_SNP_
Hanko	0.208	23.9	21.1–27.4	0.749 (0.184)
Kökar	0.187	Infinite	Infinite – infinite	0.945 (0.064)
Sastamala	0.229	7.6	7.2–8.0	0.779 (0.228)
Tohmajärvi	0.440	0.8	0.8–0.8	0.835 (0.332)
Vihti	0.184	Infinite	Infinite ‐ infinite	0.872 (0.051)
Matsalu	0.199	50.9	42.9–62.2	0.510 (0.167)
Ridala	0.202	38.9	34.7–44.1	0.442 (0.161)
Vormsi	0.102	13.2	12.8–13.5	0.593 (0.149)
Belarus	0.213	14.4	13.6–15.4	0.526 (0.123)
Poland	0.306	1.5	1.5–1.5	0.505 (0.201)
Slovenia	0.218	12.1	11.6–12.7	0.407 (0.123)

Note

Infinite *N*_e_ estimates occur when genetic variation is high and the sampling error among individuals is stronger than the signal of genetic drift from a finite number of parents (Do et al., 2014), thus infinite ≠ infinitely large population.

**Figure 3:**
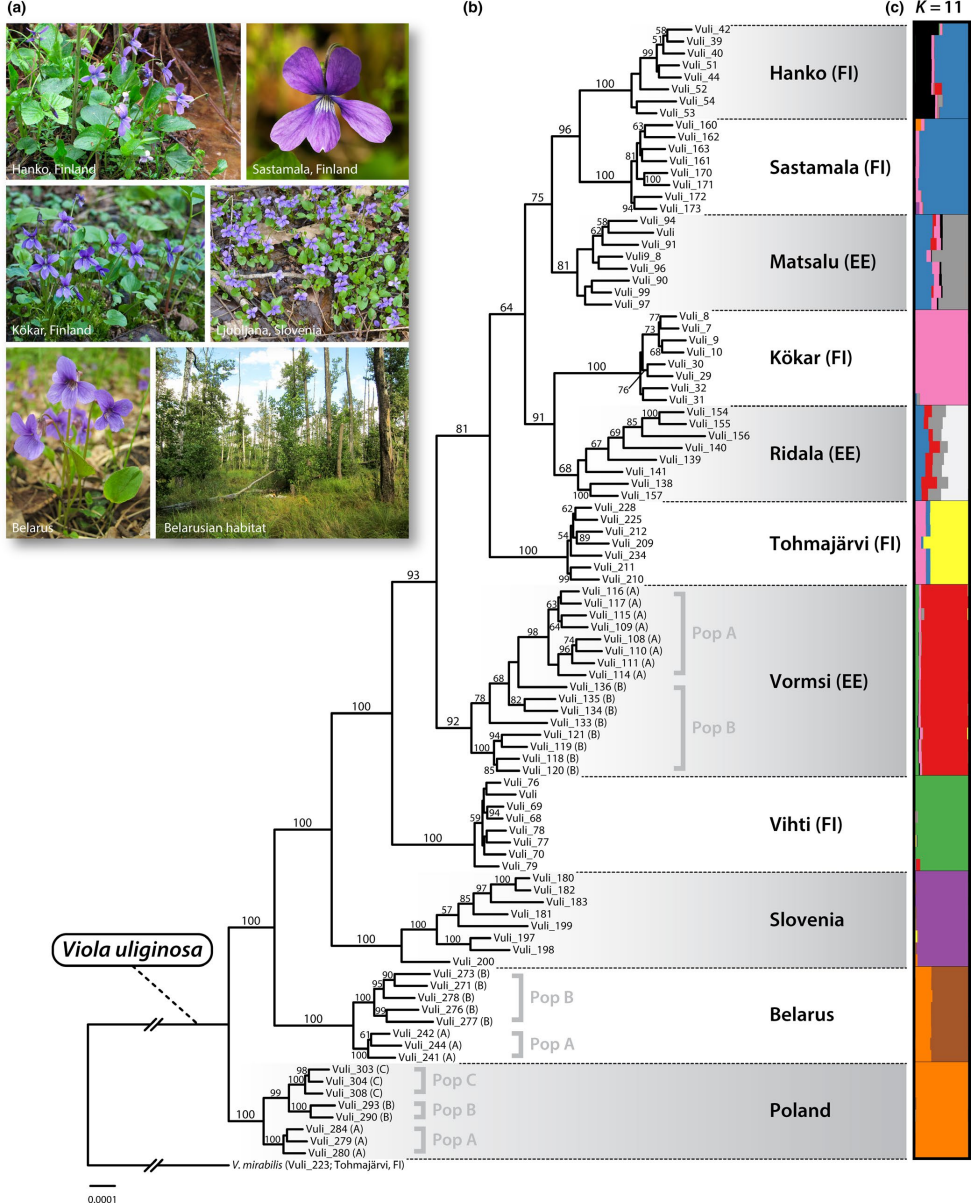
(a) Representative photographs of *Viola uliginosa*. (b) Phylogenetic relationships of *V. uliginosa* including one outgroup species, *V. mirabilis* (Vuli_223). Maximum‐likelihood tree inferred from RAxML analysis based on de novo assembly method. The data matrix consisted of 31,724 SNPs in 2,040,210 bp. The bootstrap values shown near the branches are from 1,000 rapid bootstrap resamplings. (c) The barplot, generated through Structure software, shows the assignments of individuals into 11 genetic clusters. Each bar represents one individual, and colors represent the proportion of the individuals that belong to each of the genetic cluster

The maximum‐likelihood tree (Figure 3 and Figure S2) showed that each population is monophyletic with high support and that Estonian and Finnish populations were mixed among each other. The Polish population was the closest to the root, with the Belarusian and Slovenian populations forming the next level of branching. Adjacent subpopulations in Poland and in Belarus were monophyletic and formed sister clades to subpopulations in the same locality. The subpopulations from Vormsi, Estonia, showed paraphyly. The similar topology with all 11 major clades was also recovered in the phylogenetic network (Figure S3), where the Central European populations (Poland, Slovenia, and Belarus) were separated from North European populations by a long branch and the Finnish and Estonian populations were close to each other. However, the reticulation observed in the phylogenetic network between Finnish and Estonian populations, especially in adjacent Vormsi and Ridala populations, is indicative of hybridization and/or incomplete lineage sorting.

Structure analysis (Figure 3) suggested the optimal number of estimated genetic clusters was 11, corresponding to the total number of populations studied. However, some indications of admixture were present, and specifically, individuals from Hanko (Finland, *Q* values ranged between 0.39 and 0.60), Matsalu (Estonia, *Q* values: 0.17–0.29), and Ridala (Estonia, *Q* values: 0.08–0.19) had parts of their genomes assigned to the same cluster with individuals from Sastamala (Finland). Similarly, individuals from Belarus were partially assigned to the same cluster as individuals from Poland (*Q* values: 0.26–0.32). The FineRADstructure analysis showed a clear separation of the southern (Slovenia, Poland, and Belarus) from northern (Finland and Estonia) populations and also weak structuring within the northern populations (Figure S4).

AMOVA and pairwise *F*_ST_ results further supported strong genetic differentiation of the populations. AMOVA analysis suggested that 37.29% of variation arose from differentiation among populations within groups (*F*_SC_ = 0.423) and 11.85% from differentiation among groups (*F*_CT_ = 0.119), with a total *F*_ST_ of 0.492 (Table S2). Pairwise *F*_ST_ values were high, ranging from 0.154 between Ridala and Matsalu to 0.628 between Kökar and Poland, and all had *p*‐values < .02 (Table S3). Mantel test revealed the correlation coefficient *r* = 0.471 (*p* = .023), with 22.2% of the geographic distance determined by the genetic distance.

All populations, except Ridala and Hanko, showed negative Tajima's *D* values and small raggedness with *p*‐values < .05, suggesting past population expansion (Table 2). This was further supported by the mismatch distributions constructed from all the data and from the Finnish and Estonian populations (Figure S5). The best scenario according to the DIYABC was scenario 2 in Figure 2 (simultaneous divergence of first Estonian and then Finnish populations) that received 100% support against the one constructed using the branching order of the phylogenetic tree. Based on the principal component analysis, the observed data were located among the data simulated according to scenario 2 and distant from data simulated according to scenario 1 (see Figure S6). Divergence times were given in generations, and as there were no estimates for a generation time in *V. uliginosa*, we used a mean of estimates obtained from *V. elatior*, *V. pumila,* and *V. stagnina* from Eckstein, Danihelka, and Otte (2009), 9.44 years, to transfer the generations into years (Table S4). Using this estimate, divergence from the common ancestor occurred 9,140 years BP (95% CI: 4,630–9,360; 824 generations), divergence of Estonian populations 550 years BP (95% CI: 280–1,650; 85.1 generations), and divergence of Finnish populations just 100 years BP (95% CI: 94.4–140; 11.6 generations).

## 4 DISCUSSION

### 4.1 Genetic diversity, inbreeding, and effective population size

Estimates of nucleotide diversities from our study populations (0.013–0.023) were at an intermediate level compared with previous studies on other plant species using ddRAD sequencing. For example, much lower levels were found in endangered Hawaiian lobelioid *Clermontia* (0.0014) and *Cyanea* (0.0012) populations (Jennings et al., 2016) and much higher estimates were detected in endangered Indian orchids *Geodorum densiflorum* (0.036), *Dendrobium densiflorum* (0.106), and *Rhynchostylis retusa* (0.113). Levels similar to our study were found in *Cymbidium aloifolium* (0.014) (Roy, Moitra, & Sarker, 2017). We are not aware of other studies of violets using ddRAD sequencing; however, other methods have been used for population analyses. For example, RAPD markers and population demographic methods revealed high genetic polymorphism and both clonal and sexual reproduction in *Viola riviniana* (Auge, Neuffer, Erlinghagen, Grupe, & Brandl, 2001). Gene diversity estimates from AFLP data from three violet species in Central Europe, *Viola elatior*, *V. pumila,* and *V. stagnina*, were relatively low (0.113–0.174) and varied between populations located in the central or peripheral parts of the species ranges (Eckstein, Hölzel, & Danihelka, 2006). Asian violets *Viola grayi*, *V. kusanoana,* and *V. grypoceras* showed expected microsatellite heterozygosity of 0.078–0.773, depending on the species and population, with the endangered coastal violet *V. grayi* having the lowest genetic diversity (Hirai, Kubo, Ohsako, & Utsumi, 2012). Thus, the genus *Viola* in general has wide variation in genetic diversity, likely stemming from different levels of sexual reproduction, varying population sizes, generation lengths, and ages of species and populations, and in addition to the strength and type of selection, the species and/or populations are facing. Genetic diversity of *V. uliginosa* in Poland suggested high genetic uniformity over the study populations, when AFLP markers were applied. Similar to our results, each individual possessed an own genotype profile although individuals from a given subpopulation were very alike, with similarity coefficients typically of 0.94–0.99 (Cieślak et al., 2006).

Individual inbreeding coefficients in our study populations were high, with 68 individuals from the 95 studied having values above 0.500. Similar high inbreeding coefficients have been observed in the endangered seaside violet *V. grayi* (Hirai et al., 2012). In addition, all effective population sizes for *V. uliginosa* were < 51, supporting a reproductive strategy favoring clonal propagation. The northernmost population in Finland had the highest inbreeding coefficient and the *N*_e_ estimate for one population was < 1, possibly due to a higher proportion of clonality. It has been commonly suggested that ecologically marginal populations suffer from small effective population sizes, because of fragmentation and lack of gene flow due to lack of suitable habitat (e.g., Caughley, Grice, Barker, & Brown, 1988; Young, Boyle, & Brown, 1996). The small *N*_e_ is manifested by an increase in self‐pollination, inbreeding, and clonality in plants at the edge populations when compared to central populations (Arnaud‐haond et al., 2006; Beatty, McEvoy, Sweeney, & Provan, 2008; Eckert, 2002; Silvertown, 2008). Our data support the abundant center hypothesis, which predicts that genetic diversity is lowest at the species margin because biological tolerances of a species reach their limit and/or due to repeated founder events during postglacial colonization (Brown, 1984; Petit et al., 2003; Sagarin & Gaines, 2002). However, the hypothesis is subject to further evaluation, as a meta‐analysis reported a decline in genetic diversity toward range margins in only 64.2% of the reviewed 134 studies (Eckert et al., 2010).

Another factor explaining low genetic variation, small effective population sizes, and high inbreeding coefficients, characteristic of some *V. uliginosa* populations, may be that their habitats are affected by temporal stochastic changes. In Finland, the occupied habitats are separated by 60–520 km compared with the Estonian populations at 35–63 km. Thus, if a strong environmental disturbance exposes the population to a genetic bottleneck, there is no gene flow to rescue the population. This is supported further by the high pairwise *F*_ST_ values among the Finnish populations (average 0.520) compared with Estonian populations (average 0.201) and among the eastern and southern European populations from Slovenia, Belarus, and Poland (average 0.454). The latter are separated by 530–1,890 km, yet their *F*_ST_ values were smaller than between Finnish populations. This can be explained by that occasional gene flow among the southern populations may be more likely than among the Finnish populations. Most likely *V. uliginosa* has never been common in Finland, as it requires a rare, eutrophic, and flooded habitat and thus gene flow seldom, if ever occurs. Only 17 sites have been recorded since 1851, and all but six are now extinct. The six extant populations have existed for a range of time with the oldest being Vihti (1851) and the youngest at Tohmajärvi (discovered in 1999). The present sizes of the populations vary between tens and thousands of rosettes (Ranta & Siitonen, 2011). Thus, if the populations have been founded via long‐distance dispersal by just a few seeds and/or have experienced repeated bottlenecks, they have lost genetic diversity several times in their history, simultaneously resulting in low effective population sizes and high inbreeding coefficients.

Nevertheless, some individuals displayed strikingly smaller inbreeding coefficients than others, (e.g., Tohmajärvi, Finland, 0.084; Ridala, Estonia, 0.100), strongly suggesting that occasional outbreeding reduced homozygosity of otherwise very homogeneous populations. In vitro experiments revealed that a high percentage of seeds actually germinate (Ranta et al., 2016) supporting the potential importance of sexual reproduction. Indeed, the species was reintroduced in 2014 at one site after 40 years of absence and was still flowering in 2017 (Hyvärinen, 2018; Ranta, 2014). The reintroduction used seeds collected from a private garden with plants originating from almost the same site, which had been destroyed due to construction work in 1975 (this population was not sampled for this study, Kulmala, Ryttäri, & Laaka‐Lindberg, 2016; Ranta, 2014). Thus, although sexual reproduction may not be common, it can play an important role in genetic diversity of *V. uliginosa* populations, especially at the northern edge of the species’ range.

### 4.2 Population history and the origin of the Finnish populations

The phylogenetic tree constructed using the maximum‐likelihood method shows mixing of Estonian and Finnish populations with the more southern populations located closer to the outgroup (*V. mirabilis*). However, DIYABC results strongly supported divergence of Estonian populations more or less simultaneously about 550 years BP and the Finnish populations more recently, about 100 years BP (95% CI: 94.4–140). The generation time used for these time estimates is adopted from other *Viola* species (9.44 years, calculated from Eckstein et al., 2009), which possibly is not valid for our study species. On the other hand, the estimate for the branching from the most common ancestor (9 140 years ago) fits nicely to the onset of a warmer and moister period after the Younger Dryas in Europe (e.g., Mauri, Davis, Collins, & Kaplan, 2015). The mismatch distributions, Tajima's D and raggedness values suggest past expansion for most populations and for the whole data. The time of the expansion based on τ values does not seem to vary much between populations, suggesting that all populations still carry the signs of the same past expansion. Based on the estimates of ancestral effective population sizes from DIYABC, the population sizes have indeed been thousands of individuals and at the time of divergence of Slovenian and Belarusian populations, about three times larger than in the common ancestral population.

Finnish populations are phylogenetically very close to the Estonian populations, which are also geographically close. The pattern is supported by mixing of these populations in the phylogenetic tree (Figure 3), branching of the Finnish populations from the Estonian ancestor in the DIYABC analysis (Figure 2, scenario 2), and existence of the same genetic clusters in several Estonian and Finnish populations as detected with Structure analysis (“blue,” “pink,” and “gray” clusters in Figure 3). It is likely that at the time of divergence of populations in present Estonia, some individuals dispersed to Finland, which then founded the ancestral Finnish population(s) from which the present remaining populations originated. By now, population sizes have declined in most populations, probably due to founder effects as new populations have been founded by very few individuals and due to repeated bottlenecks caused by temporal changes in the habitat. The estimates of present effective sizes vary by about 10‐fold between the two different methods (linkage disequilibrium, NeEstimator and coalescence, DIYABC), but still show basically the same trend, and Finnish populations have smaller effective population sizes than Estonian populations.

### 4.3 Conservation and reintroductions

IUCN guidelines for reintroductions state that “Founders should show characteristics based on genetic provenance, and on morphology, physiology and behaviour that are assessed as appropriate through comparison with the original or any remaining wild populations” (IUCN/SSC, 2013). Maschinski and Albrecht (2017) recently presented additional guidelines for reintroduction of rare plants stating that measuring of genetic structures should be performed before reintroductions, especially if, for example, the populations are highly fragmented and isolated or if populations have less than 50 individuals setting fruit. This number likely stems from a previously recommended minimum effective population size of 50 for the prevention of loss of fitness, which has since been amended to a value of 100 (Frankham, Bradshaw, & Brook, 2014). Here, we focused on examining the few remaining natural populations of *V. uliginosa* in Finland. Three of the existing populations can be considered abundant, each with thousands of individuals (Hanko, Kökar and Tohmajärvi, Ranta et al., 2016); however, the remaining two populations are much smaller: Sastamala with < 100 individuals and Vihti with 100s individuals (unsampled population in Mäntsälä has < 10 individuals). Irrespective of the census size, the effective population sizes are much smaller than the presently recommended 100 for the prevention of loss of fitness and particularly smaller than the recommended size of 1,000 for retaining evolutionary potential (Frankham et al., 2014). Furthermore, all Finnish populations are clearly genetically differentiated and are located as isolated patches separated by 10s and 100s of km. Even though there is evidence of past common ancestry in Finnish and Estonian populations, the observed genetic distinctiveness of all populations opposes mixing of individuals between populations. Thus, for conserving these remaining populations and considering previous success in reintroduction, we propose that seed banks from the remaining populations should be founded, cultivated ex situ, and introduced to suitable, but other sites close to the original locations. This will minimize the chance that stochastic habitat changes will eliminate whole populations. Using sites that are closely located to the source population for introduction also fulfills the IUCN guideline for genetic provenance and acts as a precaution in avoiding source populations that possibly are maladapted to the site. Usage of distant source populations may even result in outbreeding depression within original populations if they crossbreed. Founding of multiple close sites can also help in increasing or at least maintaining the effective population sizes, especially if different founder individuals are used for different sites and if there is fast diversifying selection at the sites. Furthermore, the remaining populations, as well as the newly founded sites, should be monitored annually to (a) found a population ecological study to estimate vital rates and the importance of different life stages to population growth, (b) perform population viability analyses, and (c) address any stochastic changes in the population size or in the habitat as soon as possible.

## CONFLICT OF INTEREST

None declared.

## AUTHOR CONTRIBUTIONS

L. Kvist conceived and designed the study, performed some data analysis, and drafted the manuscript; K.M. Lee carried out the molecular laboratory work, performed the bioinformatics and data analysis, and drafted the manuscript; P. Ranta, J. Saarikivi, L. Kutnar, B. Vreš, and M. Dzhus collected field samples and drafted the manuscript; and M. Mutanen conceived the study and drafted the manuscript. All authors gave final approval for publication.

## Data Availability

The demultiplexed fastq data are archived in the NCBI SRA (BioProject ID: PRJNA540749). All Supporting Information material accompanying the article is posted on Dryad Digital Repository, http://doi.org/10.5061/dryad.6v78j4c.
