# Supplementary material for: Using genomic information for management planning of an endangered perennial, Viola uliginosa
Source: Ecol Evol. 2020 Feb 17;10(5):2638–49. doi: 10.1002/ece3.6093 (PMC7069310; doi:10.1002/ece3.6093)
Supplement: Supplementary file 1 [file ECE3-10-2638-s001.docx]

**Supplemental Information for:**

**Using genomic information for management planning of an endangered perennial, *Viola uliginosa***

Kyung Min Lee, Pertti Ranta, Jarmo Saarikivi, Lado Kutnar, Branko Vreš, Maxim Dzhus, Marko Mutanen & Laura Kvist


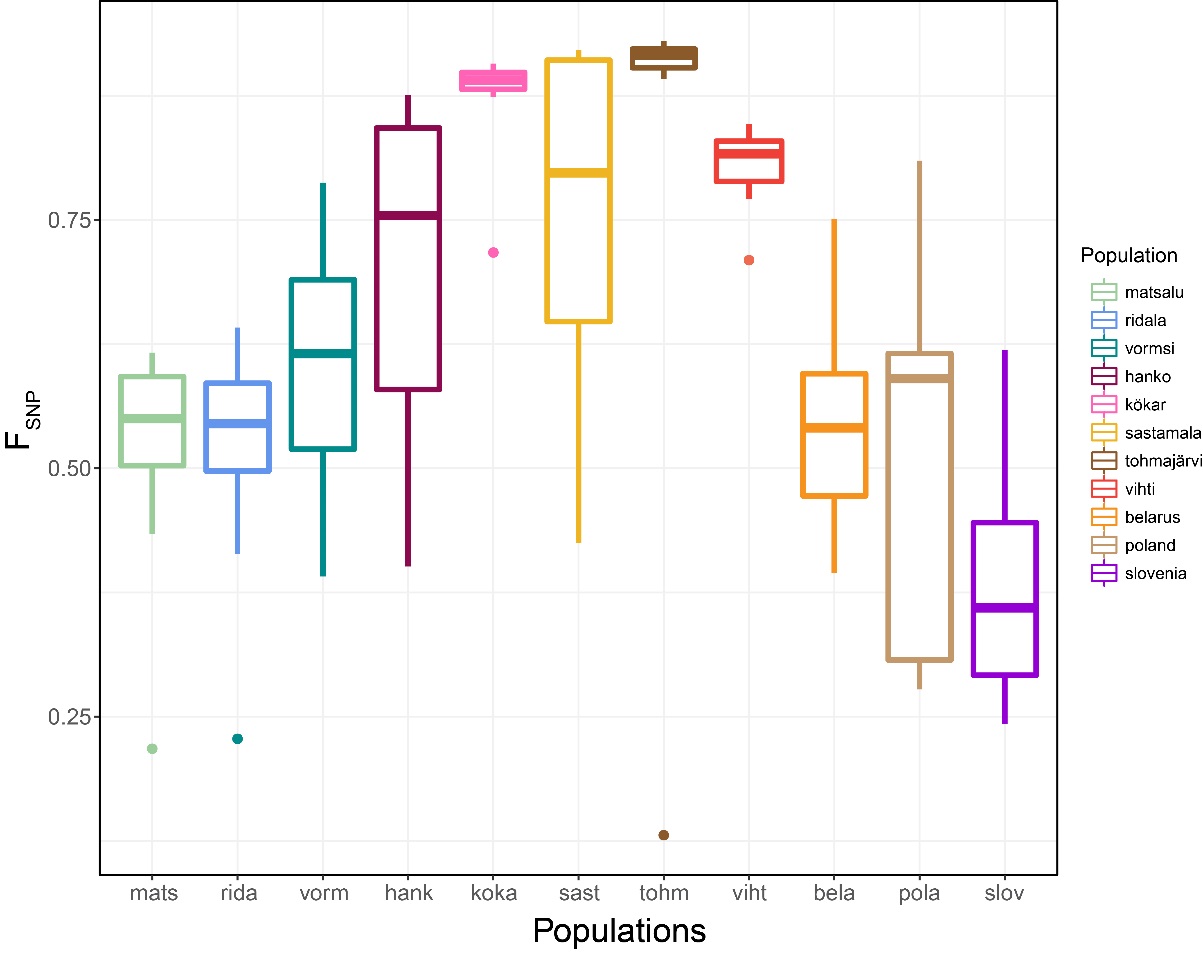


Fig. S1. Boxplot depicting F_SNP_ per population. Each boxplot represents the distribution of F_SNP_ in the all individuals sampled in each population. Population abbreviations are as in the legend.


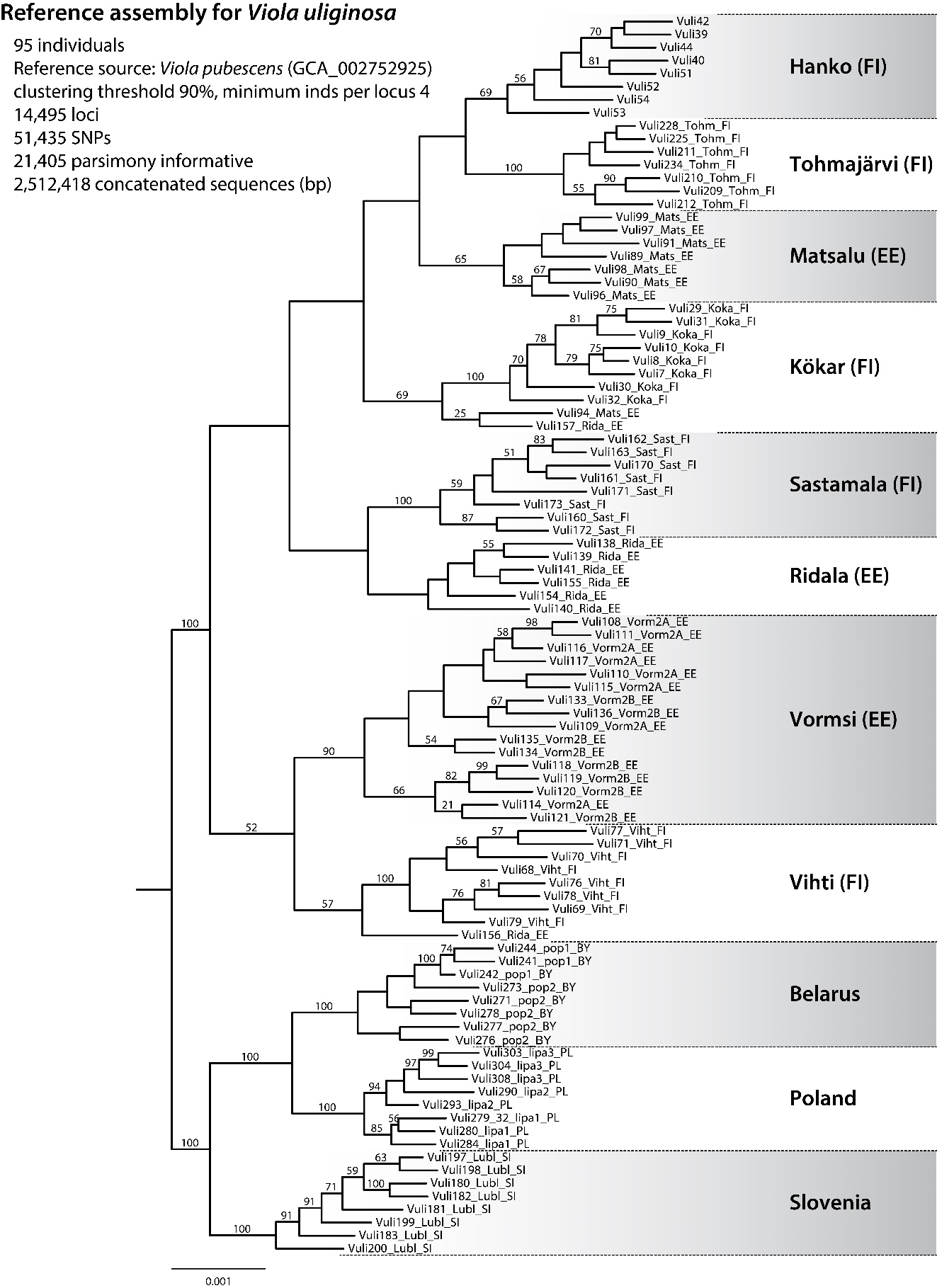


Fig. S2. Maximum likelihood tree inferred from RAxML analysis based on ddRAD data using reference assembly against *Viola pubescens* genome (GCA_002752925). The data matrix consisted of 51,435 SNPs in 2,512,418 bp. The bootstrap values shown near the branches are from 1,000 rapid bootstrap re-samplings.


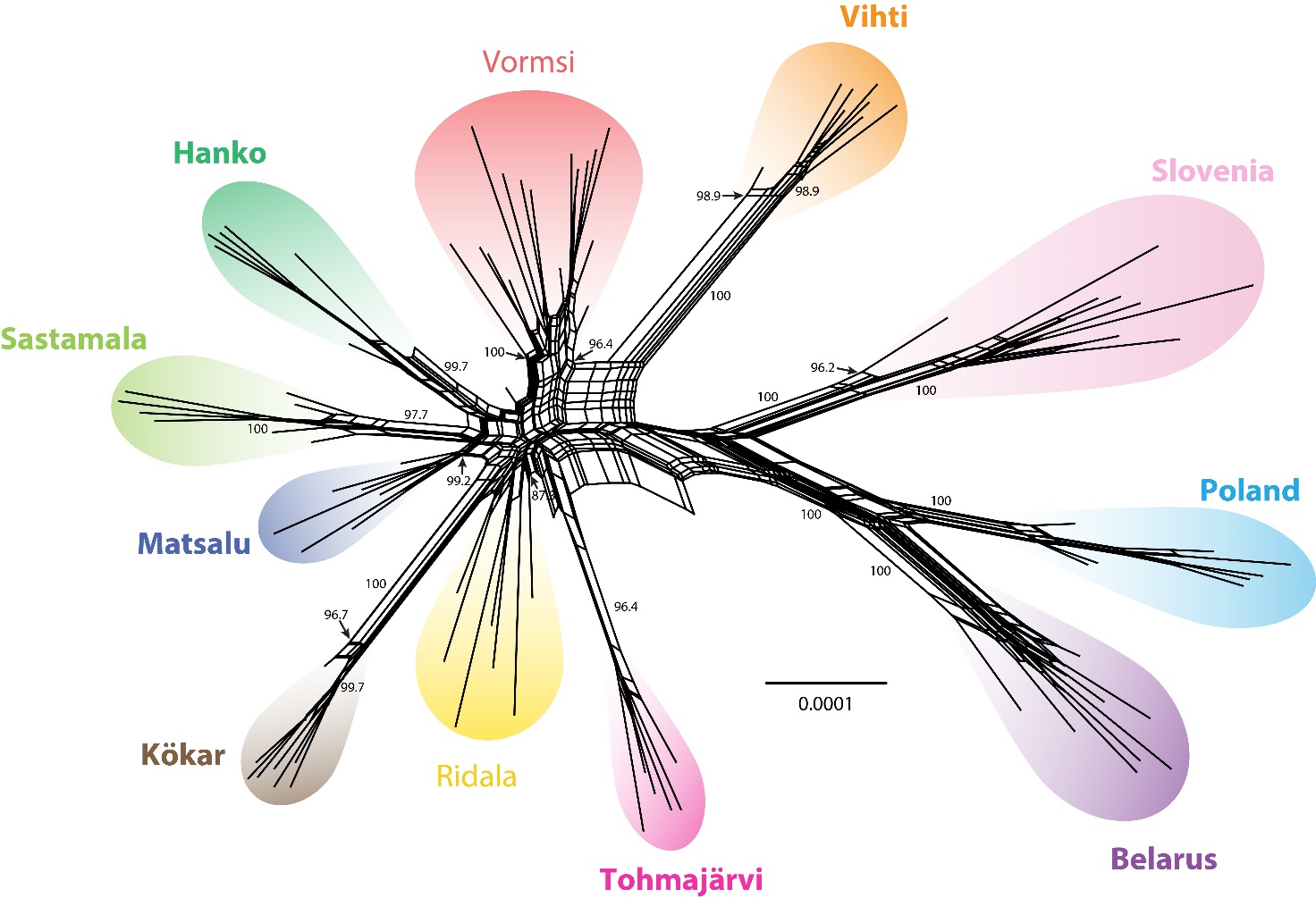


Fig. S3. Phylogenetic network of *Viola uliginosa* based on uncorrected p-distances of 31,724 SNPs from ‘ddRAD_m48’ data using SplitsTree v.4.14.2 with heterozygous ambiguities averaged and normalized. Bootstrap support values (1,000 replicates) over 75% are shown. Boxes in the network present uncertainty in the phylogeny and are expected if horizontal gene exchange has occurred or incomplete lineage sorting prevails. Text in bold represents major splits with high bootstrap values.


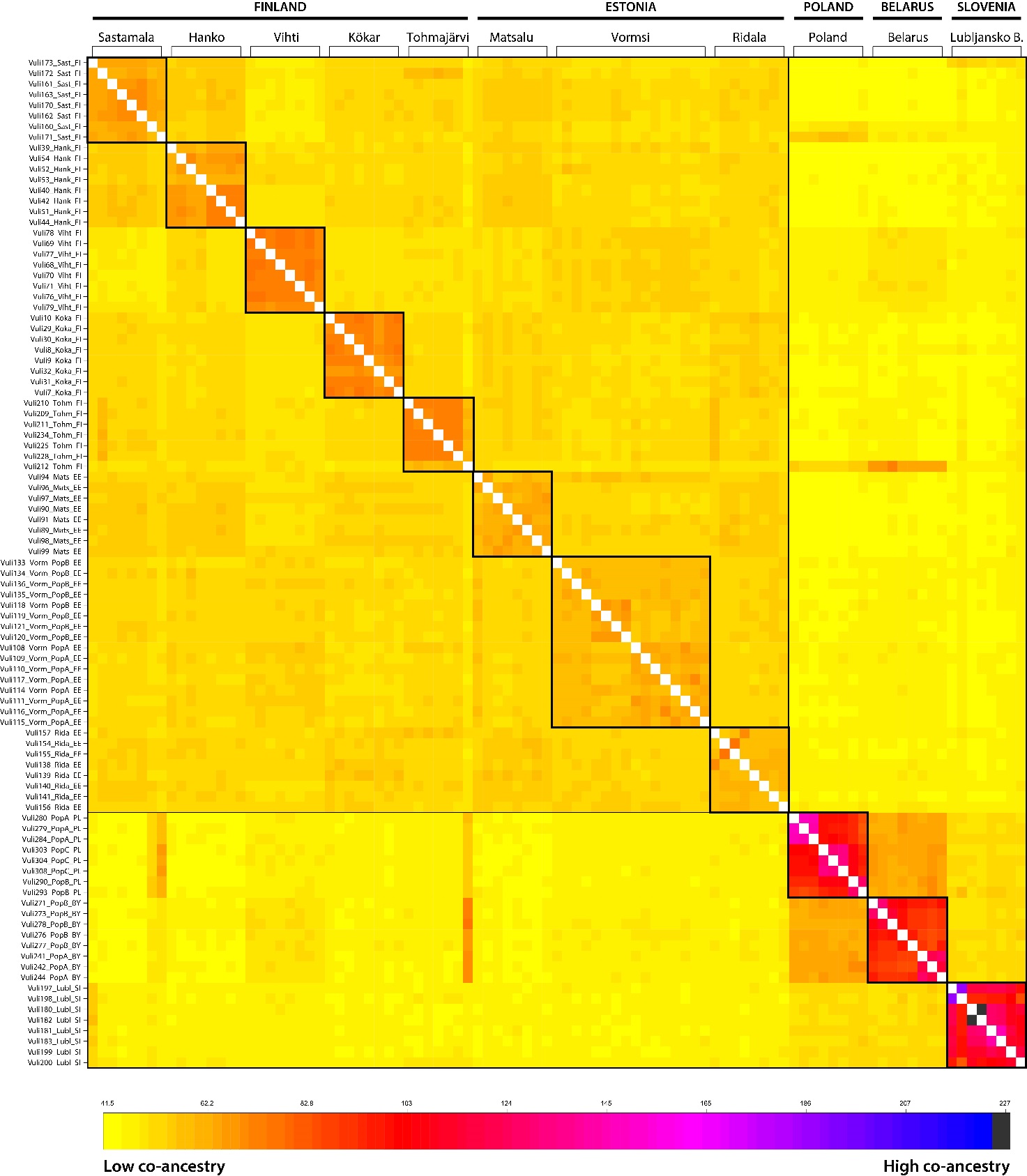


Fig. S4. Clustered FinRADstructure co-ancestry matrix for *Viola uliginosa*. The highest levels of co-ancestry are evident among individuals from Slovenian populations, indicated by black, blue and purple colors. The lowest levels of co-ancestry sharing are indicated by yellow coloration.


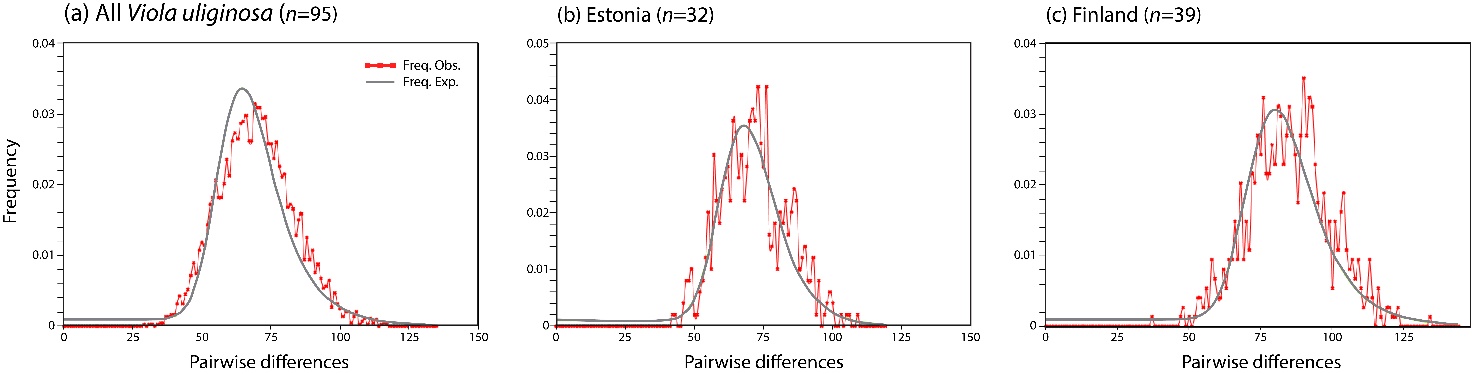


Fig. S5. The observed pairwise difference (red lines) and the expected mismatch distributions under the sudden spatial expansion model (grey lines) of the SNP dataset for *Viola uliginosa.*


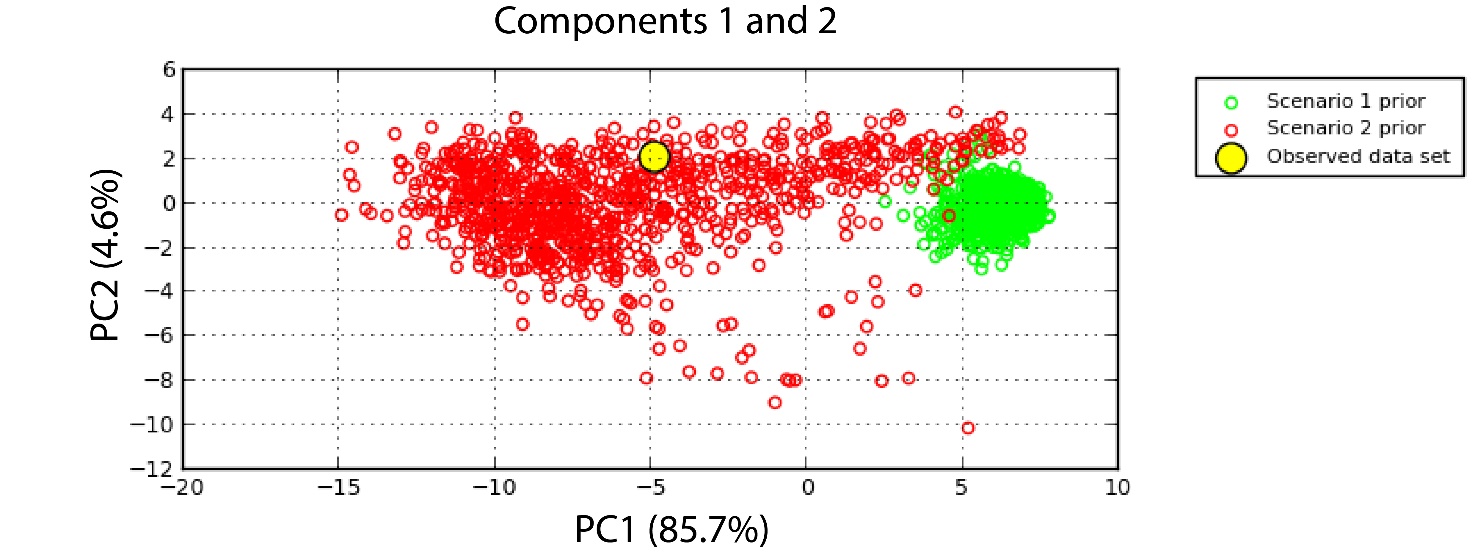


Fig. S6. The principal component analyses (PCAs) of summary statistics were performed using DIYABC v.2.0.3 software with two different scenarios based on the computer simulation of SNPs under widely predefined demographic parameters. The observed data represent summary statistics of real ddRAD SNPs data.

Appendix Table S1. Specimens of *Viola uliginosa* analysed in this study and a summary of the ddRAD data in *de novo* assembly.

| Country | Population | Sample ID | Coordinates | Collected by | Total reads (x10^6^) | Reads passed filter (x10^6^) | Clusters at 90% | Mean depth | Heterozygosity | Retained loci | Recovered loci in assembly |
| --- | --- | --- | --- | --- | --- | --- | --- | --- | --- | --- | --- |
| Belarus | Belarus Pop A | Vuli241 | 53.20 N 27.52 E | MD | 1.168 | 1.166 | 124307 | 43.67 | 0.0063 | 22239 | 9627 |
|  | Belarus Pop A | Vuli242 | 53.20 N 27.52 E | MD | 0.947 | 0.945 | 112711 | 35.90 | 0.0063 | 21095 | 9228 |
|  | Belarus Pop A | Vuli244 | 53.20 N 27.52 E | MD | 2.402 | 2.398 | 236400 | 64.39 | 0.0066 | 29581 | 9959 |
|  | Belarus Pop B | Vuli271 | 53.20 N 27.52 E | MD | 2.022 | 2.018 | 241256 | 51.91 | 0.0062 | 29883 | 9764 |
|  | Belarus Pop B | Vuli273 | 53.20 N 27.52 E | MD | 1.520 | 1.517 | 184127 | 45.61 | 0.0062 | 26472 | 9819 |
|  | Belarus Pop B | Vuli276 | 53.20 N 27.52 E | MD | 1.995 | 1.992 | 244601 | 45.19 | 0.0059 | 34783 | 10032 |
|  | Belarus Pop B | Vuli277 | 53.20 N 27.52 E | MD | 1.522 | 1.520 | 189058 | 43.98 | 0.0062 | 27015 | 9800 |
|  | Belarus Pop B | Vuli278 | 53.20 N 27.52 E | MD | 1.703 | 1.700 | 207769 | 47.98 | 0.0063 | 27800 | 9933 |
| Poland | Poland Pop A | Vuli279 | 50.42 N 22.01 E | PR | 1.123 | 1.122 | 150103 | 31.75 | 0.0063 | 27713 | 9690 |
|  | Poland Pop A | Vuli280 | 50.42 N 22.01 E | PR | 1.766 | 1.763 | 198741 | 42.96 | 0.0064 | 32077 | 9781 |
|  | Poland Pop A | Vuli284 | 50.42 N 22.01 E | PR | 1.104 | 1.102 | 125266 | 37.77 | 0.0069 | 23802 | 9815 |
|  | Poland Pop B | Vuli290 | 50.42 N 22.01 E | PR | 1.550 | 1.547 | 174508 | 46.58 | 0.0054 | 25842 | 9540 |
|  | Poland Pop B | Vuli293 | 50.42 N 22.01 E | PR | 0.656 | 0.655 | 66473 | 35.53 | 0.0040 | 14700 | 5954 |
|  | Poland Pop C | Vuli303 | 50.42 N 22.01 E | PR | 2.052 | 2.048 | 232591 | 53.17 | 0.0064 | 30122 | 9818 |
|  | Poland Pop C | Vuli304 | 50.42 N 22.01 E | PR | 1.828 | 1.825 | 180053 | 54.29 | 0.0066 | 27515 | 10012 |
|  | Poland Pop C | Vuli308 | 50.42 N 22.01 E | PR | 1.854 | 1.850 | 198989 | 53.36 | 0.0067 | 27857 | 9889 |
| Slovenia | Lubljansko Barje | Vuli180 | 46.02 N 14.51 E | BR & LK | 1.444 | 1.442 | 356052 | 14.47 | 0.0032 | 72406 | 10306 |
|  | Lubljansko Barje | Vuli181 | 46.02 N 14.51 E | BR & LK | 1.470 | 1.467 | 377427 | 11.55 | 0.0028 | 94862 | 9549 |
|  | Lubljansko Barje | Vuli182 | 46.02 N 14.51 E | BR & LK | 1.215 | 1.213 | 311409 | 12.59 | 0.0030 | 70171 | 9767 |
|  | Lubljansko Barje | Vuli183 | 46.02 N 14.51 E | BR & LK | 1.501 | 1.498 | 423298 | 10.79 | 0.0028 | 100795 | 9683 |
|  | Lubljansko Barje | Vuli197 | 46.02 N 14.51 E | BR & LK | 1.476 | 1.473 | 380919 | 13.73 | 0.0030 | 76481 | 10004 |
|  | Lubljansko Barje | Vuli198 | 46.02 N 14.51 E | BR & LK | 1.377 | 1.375 | 399981 | 10.23 | 0.0023 | 98353 | 5887 |
|  | Lubljansko Barje | Vuli199 | 46.02 N 14.51 E | BR & LK | 1.525 | 1.523 | 452766 | 9.40 | 0.0021 | 118440 | 6701 |
|  | Lubljansko Barje | Vuli200 | 46.02 N 14.51 E | BR & LK | 1.819 | 1.815 | 363353 | 17.68 | 0.0033 | 78717 | 10421 |
| Estonia | Matsalu | Vuli89 | 58.77 N 23.75 E | PR | 2.124 | 2.120 | 206812 | 61.72 | 0.0064 | 27285 | 10360 |
|  | Matsalu | Vuli90 | 58.77 N 23.75 E | PR | 2.135 | 2.131 | 231726 | 58.26 | 0.0062 | 28638 | 10262 |
|  | Matsalu | Vuli91 | 58.77 N 23.75 E | PR | 1.897 | 1.894 | 205528 | 54.80 | 0.0064 | 26867 | 10353 |
|  | Matsalu | Vuli94 | 58.77 N 23.75 E | PR | 2.882 | 2.877 | 295557 | 71.85 | 0.0066 | 30902 | 10337 |
|  | Matsalu | Vuli96 | 58.77 N 23.75 E | PR | 1.153 | 1.152 | 126845 | 40.73 | 0.0064 | 22863 | 10150 |
|  | Matsalu | Vuli97 | 58.77 N 23.75 E | PR | 0.953 | 0.951 | 119286 | 35.09 | 0.0064 | 21765 | 10102 |
|  | Matsalu | Vuli98 | 58.77 N 23.75 E | PR | 0.932 | 0.931 | 107681 | 35.60 | 0.0064 | 21144 | 9917 |
|  | Matsalu | Vuli99 | 58.77 N 23.75 E | PR | 1.063 | 1.061 | 132453 | 37.15 | 0.0063 | 22631 | 10109 |
|  | Ridala | Vuli138 | 58.93N 23.54 E | PR | 2.582 | 2.577 | 278041 | 56.92 | 0.0061 | 33968 | 9898 |
|  | Ridala | Vuli139 | 58.93N 23.54 E | PR | 1.634 | 1.631 | 169787 | 48.17 | 0.0067 | 26272 | 9813 |
|  | Ridala | Vuli140 | 58.93N 23.54 E | PR | 1.856 | 1.852 | 232678 | 45.46 | 0.0060 | 30280 | 9527 |
|  | Ridala | Vuli141 | 58.93N 23.54 E | PR | 1.117 | 1.115 | 147721 | 32.08 | 0.0056 | 27069 | 9706 |
|  | Ridala | Vuli154 | 58.93N 23.54 E | PR | 1.528 | 1.525 | 172238 | 44.82 | 0.0063 | 26756 | 10152 |
|  | Ridala | Vuli155 | 58.93N 23.54 E | PR | 1.267 | 1.265 | 157956 | 39.87 | 0.0064 | 25064 | 9876 |
|  | Ridala | Vuli156 | 58.93N 23.54 E | PR | 1.905 | 1.902 | 231006 | 45.57 | 0.0058 | 31213 | 9488 |
|  | Ridala | Vuli157 | 58.93N 23.54 E | PR | 1.838 | 1.834 | 240213 | 47.16 | 0.0061 | 29563 | 10235 |
|  | Vormsi Pop A | Vuli108 | 59.02 N 23.26 E | PR | 1.590 | 1.588 | 157097 | 52.97 | 0.0064 | 24432 | 10316 |
|  | Vormsi Pop A | Vuli109 | 59.02 N 23.26 E | PR | 2.024 | 2.021 | 211727 | 58.92 | 0.0063 | 27257 | 10352 |
|  | Vormsi Pop A | Vuli110 | 59.02 N 23.26 E | PR | 1.692 | 1.689 | 212069 | 46.67 | 0.0059 | 27583 | 10122 |
|  | Vormsi Pop A | Vuli111 | 59.02 N 23.26 E | PR | 1.564 | 1.561 | 174355 | 48.96 | 0.0064 | 25162 | 10187 |
|  | Vormsi Pop A | Vuli114 | 59.02 N 23.26 E | PR | 1.477 | 1.475 | 163438 | 50.48 | 0.0064 | 23277 | 9259 |
|  | Vormsi Pop A | Vuli115 | 59.02 N 23.26 E | PR | 1.311 | 1.308 | 171379 | 35.02 | 0.0051 | 29580 | 9105 |
|  | Vormsi Pop A | Vuli116 | 59.02 N 23.26 E | PR | 0.854 | 0.852 | 103505 | 34.97 | 0.0061 | 19441 | 8556 |
|  | Vormsi Pop A | Vuli117 | 59.02 N 23.26 E | PR | 1.518 | 1.515 | 156746 | 52.02 | 0.0061 | 23351 | 9440 |
|  | Vormsi Pop B | Vuli118 | 59.02 N 23.26 E | PR | 1.473 | 1.471 | 159776 | 50.69 | 0.0062 | 22912 | 9203 |
|  | Vormsi Pop B | Vuli119 | 59.02 N 23.26 E | PR | 1.574 | 1.571 | 172639 | 51.87 | 0.0061 | 23843 | 9364 |
|  | Vormsi Pop B | Vuli120 | 59.02 N 23.26 E | PR | 2.129 | 2.126 | 215068 | 64.68 | 0.0064 | 25698 | 9516 |
|  | Vormsi Pop B | Vuli121 | 59.02 N 23.26 E | PR | 3.081 | 3.076 | 294915 | 75.28 | 0.0062 | 31535 | 9650 |
|  | Vormsi Pop B | Vuli133 | 59.02 N 23.26 E | PR | 0.947 | 0.946 | 108607 | 37.40 | 0.0059 | 20201 | 8654 |
|  | Vormsi Pop B | Vuli134 | 59.02 N 23.26 E | PR | 1.115 | 1.113 | 127379 | 40.67 | 0.0061 | 21744 | 8941 |
|  | Vormsi Pop B | Vuli135 | 59.02 N 23.26 E | PR | 1.433 | 1.430 | 141020 | 48.56 | 0.0061 | 23635 | 9268 |
|  | Vormsi Pop B | Vuli136 | 59.02 N 23.26 E | PR | 2.341 | 2.336 | 224980 | 67.77 | 0.0063 | 26874 | 9479 |
| Finland | Hanko | Vuli39 | 59.83 N 22.97 E | PR | 1.715 | 1.712 | 368456 | 22.08 | 0.0053 | 55824 | 10047 |
|  | Hanko | Vuli40 | 59.83 N 22.97 E | PR | 1.785 | 1.782 | 239283 | 39.13 | 0.0051 | 34878 | 10027 |
|  | Hanko | Vuli42 | 59.83 N 22.97 E | PR | 1.680 | 1.676 | 189833 | 49.44 | 0.0058 | 27119 | 10031 |
|  | Hanko | Vuli44 | 59.83 N 22.97 E | PR | 1.632 | 1.630 | 282612 | 22.25 | 0.0044 | 57936 | 9723 |
|  | Hanko | Vuli51 | 59.83 N 22.97 E | PR | 1.700 | 1.697 | 245795 | 34.79 | 0.0046 | 38741 | 9559 |
|  | Hanko | Vuli52 | 59.83 N 22.97 E | PR | 1.550 | 1.546 | 231086 | 37.32 | 0.0053 | 31914 | 9842 |
|  | Hanko | Vuli53 | 59.83 N 22.97 E | PR | 1.701 | 1.698 | 281851 | 26.58 | 0.0043 | 50393 | 9762 |
|  | Hanko | Vuli54 | 59.83 N 22.97 E | PR | 1.907 | 1.904 | 294980 | 36.90 | 0.0048 | 39897 | 9533 |
|  | Kökar | Vuli10 | 59.93 N 20.89 E | PR | 1.533 | 1.530 | 181745 | 49.56 | 0.0058 | 24635 | 9891 |
|  | Kökar | Vuli29 | 59.93 N 20.89 E | PR | 2.149 | 2.145 | 261355 | 45.14 | 0.0045 | 37702 | 10076 |
|  | Kökar | Vuli30 | 59.93 N 20.89 E | PR | 1.575 | 1.573 | 181252 | 50.12 | 0.0059 | 24904 | 9980 |
|  | Kökar | Vuli31 | 59.93 N 20.89 E | PR | 1.822 | 1.819 | 201550 | 54.56 | 0.0058 | 26387 | 9976 |
|  | Kökar | Vuli32 | 59.93 N 20.89 E | PR | 1.974 | 1.971 | 226082 | 53.14 | 0.0056 | 29223 | 10257 |
|  | Kökar | Vuli7 | 59.93 N 20.89 E | PR | 2.085 | 2.081 | 331505 | 31.16 | 0.0038 | 51102 | 10026 |
|  | Kökar | Vuli8 | 59.93 N 20.89 E | PR | 1.130 | 1.128 | 155350 | 30.31 | 0.0047 | 29468 | 9605 |
|  | Kökar | Vuli9 | 59.93 N 20.89 E | PR | 1.529 | 1.526 | 231010 | 34.48 | 0.0044 | 34522 | 9924 |
|  | Sastamala | Vuli160 | 61.34 N 22.92 E | PR | 1.967 | 1.963 | 260187 | 47.85 | 0.0062 | 30933 | 10115 |
|  | Sastamala | Vuli161 | 61.34 N 22.92 E | PR | 1.731 | 1.728 | 236359 | 42.67 | 0.0060 | 30390 | 9727 |
|  | Sastamala | Vuli162 | 61.34 N 22.92 E | PR | 1.954 | 1.951 | 283724 | 37.93 | 0.0057 | 37818 | 9753 |
|  | Sastamala | Vuli163 | 61.34 N 22.92 E | PR | 1.934 | 1.931 | 304068 | 35.91 | 0.0053 | 40298 | 9966 |
|  | Sastamala | Vuli170 | 61.34 N 22.92 E | PR | 2.050 | 2.046 | 258944 | 51.60 | 0.0062 | 31234 | 10392 |
|  | Sastamala | Vuli171 | 61.34 N 22.92 E | PR | 2.376 | 2.371 | 394904 | 40.40 | 0.0052 | 43912 | 10500 |
|  | Sastamala | Vuli172 | 61.34 N 22.92 E | PR | 2.201 | 2.196 | 447689 | 29.80 | 0.0049 | 51991 | 10514 |
|  | Sastamala | Vuli173 | 61.34 N 22.92 E | PR | 1.864 | 1.861 | 621989 | 12.23 | 0.0039 | 92900 | 10303 |
|  | Tohmajärvi | Vuli209 | 62.22 N 30.32 E | JS | 2.368 | 2.363 | 292597 | 46.16 | 0.0043 | 39401 | 9914 |
|  | Tohmajärvi | Vuli210 | 62.22 N 30.32 E | JS | 2.314 | 2.310 | 264184 | 57.62 | 0.0054 | 31579 | 10154 |
|  | Tohmajärvi | Vuli211 | 62.22 N 30.32 E | JS | 2.172 | 2.168 | 306260 | 29.36 | 0.0031 | 56634 | 9768 |
|  | Tohmajärvi | Vuli212 | 62.22 N 30.32 E | JS | 3.004 | 2.999 | 385956 | 40.23 | 0.0039 | 57404 | 10186 |
|  | Tohmajärvi | Vuli225 | 62.22 N 30.32 E | JS | 1.246 | 1.244 | 168204 | 38.54 | 0.0053 | 25455 | 9723 |
|  | Tohmajärvi | Vuli228 | 62.22 N 30.32 E | JS | 0.635 | 0.634 | 116684 | 20.99 | 0.0049 | 23515 | 9020 |
|  | Tohmajärvi | Vuli234 | 62.22 N 30.32 E | JS | 1.948 | 1.945 | 288897 | 29.55 | 0.0035 | 51321 | 9996 |
|  | Vihti | Vuli68 | 60.43 N 24.32 E | PR | 1.776 | 1.772 | 223693 | 49.62 | 0.0061 | 28209 | 9739 |
|  | Vihti | Vuli69 | 60.43 N 24.32 E | PR | 1.786 | 1.783 | 265087 | 44.62 | 0.0059 | 30386 | 9210 |
|  | Vihti | Vuli70 | 60.43 N 24.32 E | PR | 1.242 | 1.240 | 161902 | 43.37 | 0.0061 | 22599 | 9058 |
|  | Vihti | Vuli71 | 60.43 N 24.32 E | PR | 2.431 | 2.427 | 291447 | 63.85 | 0.0061 | 29191 | 9369 |
|  | Vihti | Vuli76 | 60.43 N 24.32 E | PR | 1.289 | 1.287 | 239005 | 27.49 | 0.0050 | 34497 | 9034 |
|  | Vihti | Vuli77 | 60.43 N 24.32 E | PR | 1.563 | 1.560 | 188468 | 49.85 | 0.0059 | 24973 | 9360 |
|  | Vihti | Vuli78 | 60.43 N 24.32 E | PR | 1.535 | 1.533 | 198272 | 47.99 | 0.0059 | 25216 | 9259 |
|  | Vihti | Vuli79 | 60.43 N 24.32 E | PR | 1.389 | 1.387 | 208625 | 35.35 | 0.0056 | 29761 | 9438 |
|  | **Outgroup (*V. mirabilis)*** | Vuli223 | 62.22 N 30.32 E | JS | 0.347 | 0.346 | 80902 | 11.13 | 0.0089 | 22918 | 3850 |
|  |  | **AVERAGE** |  |  | **1.673** | **1.670** | **230981** | **41.23** | **0.0055** | **35987** | **9618** |

MD = Maxim Dzhus, LK = Lado Kutnar, PR = Pertti Ranta, JS = Jarmo Saarikivi, BV = Branko Vreš

Permit numbers for sampling: 35601-48/2018-5, POKELY/155/07.01.2013, PIRELY/3325/2014, PIRELY/3584/2015, 15.6.2015, UUDELY/318/07.01/2013.

Appendix Table S2. Analysis of Molecular Variance (AMOVA) for *Viola uliginosa* based on 31,724 SNPs from ‘ddRAD_m48’ data shared among all sampled localities. Groups were 1) Finnish populations, 2) Estonian populations 3) Belarus, 4) Slovenia and 5) Poland.

| Source of variation | d.f. | Sum of squares | Variance components | % of variation | Fixation indices |
| --- | --- | --- | --- | --- | --- |
| Among groups | 4 | 5994.99 | 31.22 | 11.85 | F_CT_ = 0.1185 * |
| Among populations within groups | 6 | 5829.93 | 98.21 | 37.29 | F_SC_ = **0.4231 **** |
| Within populations | 84 | 11250.20 | 133.93 | 50.85 | F_ST_ = **0.4915 **** |
| **TOTAL** |  | 23075.12 | 263.36 |  |  |

Note: Significant *P* values are indicated by * *P* < 0.05; ** and bold text: *P* < 0.02.

Appendix Table S3. Pairwise F_ST_ estimates, calculated on called genotypes of the SNP data from ‘ddRAD_m48’ data, between populations of *Viola uliginosa*.

| Population | Belarus | Poland | Slovenia | Matsalu | Ridala | Vormsi | Hanko | Kökar | Sastamala | Tohmajärvi |
| --- | --- | --- | --- | --- | --- | --- | --- | --- | --- | --- |
| Poland | 0.42050 |  |  |  |  |  |  |  |  |  |
| Slovenia | 0.47642 | 0.46494 |  |  |  |  |  |  |  |  |
| Matsalu | 0.55450 | 0.56472 | 0.47614 |  |  |  |  |  |  |  |
| Ridala | 0.48110 | 0.49668 | 0.41262 | 0.15430 |  |  |  |  |  |  |
| Vormsi | 0.51504 | 0.53324 | 0.46825 | 0.23126 | 0.21604 |  |  |  |  |  |
| Hanko | 0.59326 | 0.60100 | 0.51069 | 0.27451 | 0.29189 | 0.35985 |  |  |  |  |
| Kökar | 0.62011 | 0.62829 | 0.56618 | 0.40015 | 0.33152 | 0.44870 | 0.51974 |  |  |  |
| Sastamala | 0.60679 | 0.59876 | 0.51358 | 0.28145 | 0.28480 | 0.37181 | 0.37681 | 0.50509 |  |  |
| Tohmajärvi | 0.56329 | 0.58637 | 0.51192 | 0.34558 | 0.31980 | 0.40582 | 0.45916 | 0.53540 | 0.46002 |  |
| Vihti | 0.57022 | 0.59931 | 0.53926 | 0.48992 | 0.42438 | 0.41770 | 0.54683 | 0.62060 | 0.60261 | 0.57818 |

Note: All values have *p* < 0.02 from 1 000 permutations.

Appendix Table S4. Parameter values obtained from the DIYABC analysis. 95 % HPD = 95 % highest posterior density.

| **Parameter** | **Population** | **mean** | **median** | **mode** | **95% HPD** |
| --- | --- | --- | --- | --- | --- |
| Ne | Hanko | 70.8 | 72.7 | 72.7 | 31.6-97.3 |
|  | Kökar | 65.8 | 67.6 | 72.2 | 29.9-95.6 |
|  | Sastamala | 78.8 | 80.9 | 91.4 | 42.4-98.3 |
|  | Tohmajärvi | 27.8 | 24.1 | 19.9 | 6.66-73.0 |
|  | Vihti | 40.6 | 37.3 | 31.4 | 11.6-85.3 |
|  | Matsalu | 608 | 629 | 699 | 211-956 |
|  | Ridala | 500 | 484 | 418 | 151-924 |
|  | Vormsi | 812 | 839 | 867 | 489-983 |
|  | Belarus | 67.3 | 75.9 | 100 | 7.54-99.6 |
|  | Poland | 1460 | 1080 | 697 | 404-5830 |
|  | Slovenia | 2240 | 1760 | 977 | 512-7390 |
|  | Finnish common ancestral | 4830 | 4530 | 2740 | 763-9610 |
|  | Estonian common ancestral | 5270 | 5190 | 5600 | 1050-9730 |
|  | Common ancestral | 3120 | 3110 | 3090 | 2030-4180 |
|  | Ancestral before branching of Slovenia | 7160 | 7630 | 9260 | 22009880 |
|  | Ancestral before branching of Belarus | 6690 | 7170 | 9260 | 1350-9860 |
| Divergence time (in generations) | |  |  |  |  |
| t1 | Divergence of Finnish populations | 11.6 | 11.3 | 11.0 | 10.0-15.0 |
| t2 | Divergence of Estonian populations and Finland | 85.1 | 78.5 | 58.7 | 30.1-175 |
| t3 | Divergence of Slovenia from the rest | 704 | 720 | 738 | 405-924 |
| t4 | Divergence of Belarus from the rest | 760 | 785 | 868 | 446-962 |
| t5 | Common ancestral, divergence of Poland from the rest | 824 | 855 | 968 | 490-992 |
